# I_8_Sb_10_Ge_36_

**DOI:** 10.1107/S1600536810017496

**Published:** 2010-05-19

**Authors:** Mohammed Kars, Thierry Roisnel, Vincent Dorcet, Allaoua Rebbah, L. Carlos Otero-Diáz

**Affiliations:** a Université Houari-Boumedienne, Faculté de Chimie, Laboratoire Sciences des Matériaux, BP 32, El-Alia Bab-Ezzouar, Algérie; b Centre de Diffractométrie X, Sciences Chimiques de Rennes, UMR 6226 CNRS Université de Rennes 1, Campus de Beaulieu, Avenue du Général Leclerc, Rennes, France; c Departomento Inorgánica, Facultad C.C. Químicas, Universidad Complutense, 28040 Madrid, Espagne

## Abstract

Single crystals of the title compound, octa­iodide deca­anti­monate hexa­tria­conta­germanide, were grown by chemical transport reactions. The structure is isotypic with the analogous clathrates-I. In this structure, the (Ge,Sb)_46_ framework consists of statistically occupied Ge and Sb sites that atoms form bonds in a distorted tetra­hedral arrangement. They form polyhedra that are covalently bonded to each other by shared faces. There are two polyhedra of different sizes, *viz.* a (Ge,Sb)_20_ dodeca­hedron and a (Ge,Sb)_24_ tetra­cosa­hedron in a 1:3 ratio. The guest atom (iodine) resides inside these polyhedra with symmetry *m*3 (Wyckoff position 2*a*) and 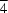
2*m* (Wyckoff position 2*d*), respectively.

## Littérature associée

La synthèse en phase vapeur des premiers clathrates *X*_8_Ge_38_ *A*_8_ (*X* = Cl, Br, I; *A* = P, As, Sb) etait décrite par Menke & von Schnering (1973 ▶) et von Schnering & Menke (1976 ▶). Les structures sont isotypes aux hydrates de gaz correspondants (Pauling & Marsh, 1952 ▶). Pour les propriétés semiconductrices et thermoélectriques, voir respectivement Chu *et al.* (1982 ▶) et Kishimoto *et al.* (2006 ▶). Pour les propriétés structurales et la conductivité thermique, voir Nolas *et al.* (2000 ▶) et Shimizu *et al.* (2009 ▶). L’histoire et les développements récents des composés type clathrate du silicium et des éléments de la colonne 14 ont été relatés par Cros & Pouchard (2009 ▶). L’étude par diffraction électronique et HRTEM du clathrate I_8_Ge_40.0_Te_5.3_ a été réalisée par Kovnir *et al.* (2006 ▶).  Pour autres composés type clathrate du germanium, voir Ayouz (2009 ▶); Latturner *et al.* (2000 ▶); Nesper *et al.* (1986 ▶).

## Partie expérimentale

### 

#### Données crystallines

I_8_Sb_10_Ge_36_
*M* *_r_* = 4834,8
Cubique, 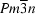

*a* = 10,8907 (2) Å
*V* = 1291,72 (3) Å^3^
*Z* = 1
Mo *K*α radiation
μ = 30,49 mm^−1^
*T* = 150 K
0,10 × 0,08 × 0,07 mm

#### Collection des données

Diffractomètre Bruker APEXII
Correction d’absorption: multi-scan (*SADABS*; Sheldrick, 2002 ▶) *T*_min_ = 0,082, *T*_max_ = 0,116
27124 réflexions mesurées
1003 réflexions indépendantes
900 réflexions avec *I* > 3σ(*I*)
*R*_int_ = 0,041

#### Affinement

*R*[*F* ^2^ > 2σ(*F* ^2^)] = 0,037
*wR*(*F* ^2^) = 0,091
*S* = 2,00
1003 réflexions
18 paramètres
3 restraintes
Δρ_max_ = 1,48 e Å^−3^
Δρ_min_ = −2,97 e Å^−3^

### 

Collection des données: *SAINT* (Bruker, 2002 ▶); affinement des paramètres de la maille: *SAINT*; reduction des données: *SAINT*; programme(s) pour la solution de la structure: *SHELXS97* (Sheldrick, 2008 ▶); programme(s) pour l’affinement de la structure: *JANA2000* (Petříček *et al.*, 2000 ▶); graphisme moléculaire: *DIAMOND* (Brandenburg & Putz, 2009 ▶); logiciel utilisé pour préparer le matériel pour publication: *JANA2000*.

## Supplementary Material

Crystal structure: contains datablocks global, I. DOI: 10.1107/S1600536810017496/br2144sup1.cif

Structure factors: contains datablocks I. DOI: 10.1107/S1600536810017496/br2144Isup2.hkl

Additional supplementary materials:  crystallographic information; 3D view; checkCIF report

## Figures and Tables

**Fig. 1.**
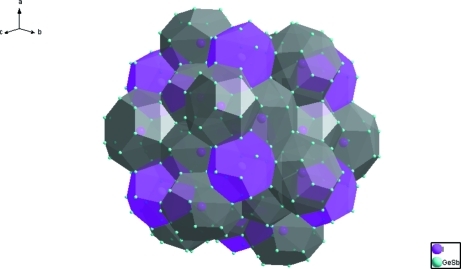
Structure du clathrate I8Sb10Ge36 montrant les deux types de polyèdres: les dodécaèdres pentagonaux (Ge, Sb)20 (en rose) et les tétrakaïdècaèdres (Ge, Sb)24 (en gris). Les atomes d'iode sont localisés au centre de ces polyèdres.

**Fig. 2.**
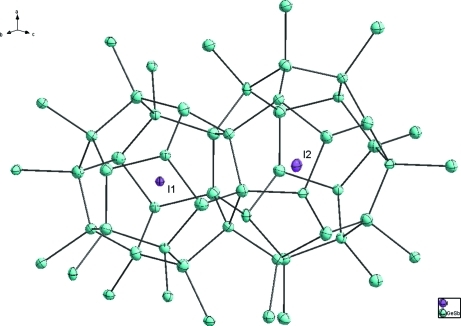
Deux cages adjacentes dans la structure du clathrate I8Sb10Ge36 avec un déplacement des ellipsoïdes à 95% de probabilité.

## supplementary crystallographic information

### Comment

Les clathrates semiconducteurs de formulation *X*_8_Ge_38_A_8_ (*X*: Cl, Br, I; A: P, As, Sb) (Menke & von Schnering, 1973; von Schnering & Menke, 1976) constituent les premiers clathrates à sous réseau hôte anionique et à réseau d'accueil mixte et cationique. Les structures de ces clathrates ont été déterminées par isotypie aux hydrates de gaz correspondants (Pauling & Marsh, 1952). De plus au cours d'essais de synthèse du clathrate vide Ge_46_, une phase qui correspond à I_8_Ge_46-_*_x_*I*_x_* (*x* = 8/3) avait été obtenue de manière inattendue (Nesper *et al.*, 1986). Ces composés ont connus un regain d'intérêt au cours de ces dernières années, essentiellement en raison de leurs propriétés semi-conductrices (Chu *et al.*, 1982), thermoélectriques (Kishimoto *et al.*, 2006) et de conductivités thermique très prometteuses (Shimizu *et al.*, 2009). L'histoire et les derniers développements des composés type clathrate du silicium et des éléments apparentés de la colonne 14 ont été relatés récement dans un étendu article par Cros & Pouchard (2009). Le composé I_8_Sb_10_Ge_36_ est décrit par la juxtaposition de deux types de polyèdres: les dodécaèdres pentagonaux (Ge, Sb)_20_ et les tétrakaïdècaèdres (Ge, Sb)_24_. Les atomes d'iode se logent au centre des cavités formées par ces polyèdres. Les atomes clathrands possédent une coordination tétraédriques avec des distances [2.5032 (3)–2.5562 (6) Å] comparables à celles obtenues dans les composés clathrates Ge_14_Ga_12_Sb_20_I_8_ [2.5792 (4)–2.6836 (3) Å] (von Schnering & Menke, 1976) et dans Ba_8_Ga_17.134_Sb_2.78_Ge_25.595_ [2.462 (3)–2.5791 (3) Å] (Latturner *et al.*, 2000). Enfin, il faut remarquer que comme pour le composé I_8_As_21_Ge_25_ (Ayouz *et al.*, 2009) l'agitation thermique (ADP's) autour de l'atome I2 (site 6*d*) est comparable à celles des autres atomes constituants le clathrate, ce n'est pas le cas de nombreux composés clathrates au germanium où l'agitation thermique autour de *X*2 est beaucoup plus large: *X*_8_Ga_16_Ge_30_ (*X*: Sr, Eu) (Nolas *et al.*, 2000) et Ge_40.0_Te_5.3_I_8_ (Kovnir *et al.*, 2006).

### Experimental

La synthèse de monocristaux de I_8_Sb_10_Ge_36_ a été réalisée par la méthode de transport en phase vapeur à partir d'un mélange d'éléments purs. Le mélange broyé puis scellé dans un tube en quartz, est porté à une température de 1100 K pendant environ une semaine.

### Refinement

La structure a été déterminée par isotypie aux clathrates-I dans le groupe d'espace *Pm*3*n* avec une occupation statistique des sites 6*c*, 16*i* et 24*k* par les atomes de germanium et d'antimoine. Tous les sites mixtes Ge/Sb ont été affinés avec une contrainte d'occupation totale égale à l'unité. Les sites 2*a* et 6*d* sont occupés totalement par les atomes d'iode. La composition du clathrate obtenue en fin d'affinement I_8_Sb_9.77_Ge_36.23_ [Ge(at.%) = 67.09; Sb(at.%) = 18.09; I(at.%) = 14.81] est proche de celle déduite par analyse chimique au MEB [Ge(at.%) = 65.19; Sb(at.%) = 18.88; I(at.%) = 15.93].

### Crystal data

**Table:**

I_8_Sb_10_Ge_36_	*D*_x_ = 6.213 Mg m^−^^3^
*M**_r_* = 4834.8	Mo *K*α radiation, λ = 0.71073 Å
Cubic, *P**m*3*n*	Cell parameters from 6120 reflections
Hall symbol: -P 4n 2 3	θ = 2.6–45.3°
*a* = 10.8907 (2) Å	µ = 30.49 mm^−^^1^
*V* = 1291.72 (3) Å^3^	*T* = 150 K
*Z* = 1	Cubic, black
*F*(000) = 2082	0.10 × 0.08 × 0.07 mm

### Data collection

**Table:**

Bruker APEXII diffractometer	1003 independent reflections
Radiation source: fine-focus sealed tube	900 reflections with *I* > 3σ(*I*)
graphite	*R*_int_ = 0.041
CCD rotation images, thin slices scans	θ_max_ = 45.3°, θ_min_ = 2.6°
Absorption correction: multi-scan (*SADABS*; Sheldrick, 2002)	*h* = −21→18
*T*_min_ = 0.082, *T*_max_ = 0.116	*k* = −21→21
27124 measured reflections	*l* = −20→20

### Refinement

**Table:**

Refinement on *F*	3 restraints
*R*[*F*^2^ > 2σ(*F*^2^)] = 0.037	Weighting scheme based on measured s.u.'s *w* = 1/[σ^2^(*F*) + 0.001444*F*^2^]
*wR*(*F*^2^) = 0.091	(Δ/σ)_max_ = 0.0004
*S* = 2.00	Δρ_max_ = 1.48 e Å^−^^3^
1003 reflections	Δρ_min_ = −2.97 e Å^−^^3^
18 parameters	

### Special details

**Table:**

Refinement. Refinement of *F*^2^ against ALL reflections. The weighted *R*-factor wR and goodness of fit *S* are based on *F*^2^, conventional *R*-factors are based on *F*, with *F* set to zero for negative *F*^2^. The threshold expression of *F*^2^ > n*σ(*F*^2^) is used only for calculating *R*-factors etc. and is not relevant to the choice of reflections for refinement. The program used for refinement, Jana2000, uses the weighting scheme based on the experimental expectations, see _refine_ls_weighting_details, that does not force *S* to be one. Therefore the values of *S* are usually larger then the ones from the SHELX program.

### Fractional atomic coordinates and isotropic or equivalent isotropic displacement parameters (Å^2^)

**Table:**

	*x*	*y*	*z*	*U*_iso_*/*U*_eq_	Occ. (<1)
I1	0	0	0	0.00492 (8)	
I2	0.25	0.5	0	0.00789 (9)	
Ge1	0.11736 (4)	0	0.30899 (4)	0.00934 (11)	0.823 (7)
Ge2	0.18365 (2)	0.18365 (2)	0.18365 (2)	0.00749 (8)	0.699 (8)
Sb3	0.25	0	0.5	0.00867 (17)	0.118 (11)
Sb1	0.117356	0	0.308994	0.00934 (11)	0.177 (7)
Sb2	0.18365	0.18365	0.18365	0.00749 (8)	0.301 (8)
Ge3	0.25	0	0.5	0.00867 (17)	0.882 (11)

### Atomic displacement parameters (Å^2^)

**Table:**

	*U*^11^	*U*^22^	*U*^33^	*U*^12^	*U*^13^	*U*^23^
I1	0.00492 (14)	0.00492 (14)	0.00492 (14)	0	0	0
I2	0.00455 (18)	0.00956 (15)	0.00956 (15)	0	0	0
Ge1	0.00836 (18)	0.00909 (18)	0.01058 (19)	0	−0.00110 (11)	0
Ge2	0.00749 (14)	0.00749 (14)	0.00749 (14)	0.00030 (7)	0.00030 (7)	0.00030 (7)
Sb3	0.0117 (4)	0.0071 (3)	0.0071 (3)	0	0	0
Sb1	0.00836 (18)	0.00909 (18)	0.01058 (19)	0	−0.00110 (11)	0
Sb2	0.00749 (14)	0.00749 (14)	0.00749 (14)	0.00030 (7)	0.00030 (7)	0.00030 (7)
Ge3	0.0117 (4)	0.0071 (3)	0.0071 (3)	0	0	0

### Geometric parameters (Å)

**Table:**

I1—Ge1	3.5997 (4)	I2—Ge1^xix^	3.6570 (3)
I1—Ge1^i^	3.5997 (4)	I2—Ge1^viii^	3.6570 (3)
I1—Ge1^ii^	3.5997 (4)	I2—Ge1^xx^	3.6570 (3)
I1—Ge1^iii^	3.5997 (4)	I2—Ge1^xxi^	3.6570 (3)
I1—Ge1^iv^	3.5997 (4)	I2—Ge1^xi^	3.6570 (3)
I1—Ge1^v^	3.5997 (4)	I2—Sb3^xvi^	3.8504
I1—Ge1^vi^	3.5997 (4)	I2—Sb3^xvii^	3.8504
I1—Ge1^vii^	3.5997 (4)	I2—Sb3^xxii^	3.8504
I1—Ge1^viii^	3.5997 (4)	I2—Sb3^xxiii^	3.8504
I1—Ge1^ix^	3.5997 (4)	I2—Sb1^xvi^	3.657
I1—Ge1^x^	3.5997 (4)	I2—Sb1^xvii^	3.657
I1—Ge1^xi^	3.5997 (4)	I2—Sb1^xviii^	3.657
I1—Ge2	3.4642 (3)	I2—Sb1^xix^	3.657
I1—Ge2^i^	3.4642 (3)	I2—Sb1^viii^	3.657
I1—Ge2^ii^	3.4642 (3)	I2—Sb1^xx^	3.657
I1—Ge2^iii^	3.4642 (3)	I2—Sb1^xxi^	3.657
I1—Ge2^xii^	3.4642 (3)	I2—Sb1^xi^	3.657
I1—Ge2^xiii^	3.4642 (3)	I2—Ge3^xvi^	3.8504
I1—Ge2^xiv^	3.4642 (3)	I2—Ge3^xvii^	3.8504
I1—Ge2^xv^	3.4642 (3)	I2—Ge3^xxii^	3.8504
I1—Sb1	3.5997	I2—Ge3^xxiii^	3.8504
I1—Sb1^i^	3.5997	Ge1—Ge1^iii^	2.5562 (6)
I1—Sb1^ii^	3.5997	Ge1—Ge2	2.5269 (4)
I1—Sb1^iii^	3.5997	Ge1—Ge2^xv^	2.5269 (4)
I1—Sb1^iv^	3.5997	Ge1—Sb3	2.5326 (4)
I1—Sb1^v^	3.5997	Ge1—Sb1^iii^	2.5562 (4)
I1—Sb1^vi^	3.5997	Ge1—Sb2	2.5269 (3)
I1—Sb1^vii^	3.5997	Ge1—Sb2^xv^	2.5269 (3)
I1—Sb1^viii^	3.5997	Ge1—Ge3	2.5326 (4)
I1—Sb1^ix^	3.5997	Ge2—Ge2^xxiv^	2.5032 (4)
I1—Sb1^x^	3.5997	Ge2—Sb1	2.5269 (3)
I1—Sb1^xi^	3.5997	Ge2—Sb1^iv^	2.5269 (3)
I1—Sb2	3.4642	Ge2—Sb1^viii^	2.5269 (3)
I1—Sb2^i^	3.4642	Ge2—Sb2^xxiv^	2.5032 (3)
I1—Sb2^ii^	3.4642	Sb3—Sb1	2.5326
I1—Sb2^iii^	3.4642	Sb3—Sb1^xxv^	2.5326
I1—Sb2^xii^	3.4642	Sb3—Sb1^xxvi^	2.5326
I1—Sb2^xiii^	3.4642	Sb3—Sb1^xxvii^	2.5326
I1—Sb2^xiv^	3.4642	Sb1—Sb1^iii^	2.5562
I1—Sb2^xv^	3.4642	Sb1—Sb2	2.5269
I2—Ge1^xvi^	3.6570 (3)	Sb1—Sb2^xv^	2.5269
I2—Ge1^xvii^	3.6570 (3)	Sb1—Ge3	2.5326
I2—Ge1^xviii^	3.6570 (3)	Sb2—Sb2^xxiv^	2.5032

Symmetry codes: (i) −*x*, −*y*, −*z*; (ii) *x*, −*y*, −*z*; (iii) −*x*, *y*, *z*; (iv) *z*, *x*, *y*; (v) −*z*, −*x*, −*y*; (vi) *z*, −*x*, −*y*; (vii) −*z*, *x*, *y*; (viii) *y*, *z*, *x*; (ix) −*y*, −*z*, −*x*; (x) −*y*, −*z*, *x*; (xi) *y*, *z*, −*x*; (xii) −*x*, −*y*, *z*; (xiii) *x*, *y*, −*z*; (xiv) −*x*, *y*, −*z*; (xv) *x*, −*y*, *z*; (xvi) −*y*+1/2, *x*+1/2, *z*−1/2; (xvii) *y*+1/2, −*x*+1/2, −*z*+1/2; (xviii) *y*+1/2, *x*+1/2, −*z*+1/2; (xix) −*y*+1/2, −*x*+1/2, *z*−1/2; (xx) −*y*, −*z*+1, −*x*; (xxi) −*y*, −*z*+1, *x*; (xxii) *z*−1/2, −*y*+1/2, *x*−1/2; (xxiii) −*z*+1/2, *y*+1/2, −*x*+1/2; (xxiv) −*y*+1/2, −*x*+1/2, −*z*+1/2; (xxv) *x*, −*y*, −*z*+1; (xxvi) −*x*+1/2, *z*−1/2, *y*+1/2; (xxvii) −*x*+1/2, −*z*+1/2, *y*+1/2.
